# Out of Sight, Out of Mind: The Effect of the Equilibration Protocol on the Structural Ensembles of Charged Glycolipid Bilayers

**DOI:** 10.3390/molecules25215120

**Published:** 2020-11-04

**Authors:** Andresa Messias, Denys E. S. Santos, Frederico J. S. Pontes, Filipe S. Lima, Thereza A. Soares

**Affiliations:** Departamento de Química Fundamental, Universidade Federal de Pernambuco, Cidade Universitária, 50670-901 Recife, Brazil; andresamessias4@gmail.com (A.M.); ewerton.denys@gmail.com (D.E.S.S.); pontesfrederico@gmail.com (F.J.S.P.); filipesilvalima@gmail.com (F.S.L.)

**Keywords:** barostat effect, GROMACS, GROMOS force field, bacterial outer membranes, lipopolysaccharide (LPS), lipid-A

## Abstract

Molecular dynamics (MD) simulations represent an essential tool in the toolbox of modern chemistry, enabling the prediction of experimental observables for a variety of chemical systems and processes and majorly impacting the study of biological membranes. However, the chemical diversity of complex lipids beyond phospholipids brings new challenges to well-established protocols used in MD simulations of soft matter and requires continuous assessment to ensure simulation reproducibility and minimize unphysical behavior. Lipopolysaccharides (LPS) are highly charged glycolipids whose aggregation in a lamellar arrangement requires the binding of numerous cations to oppositely charged groups deep inside the membrane. The delicate balance between the fully hydrated carbohydrate region and the smaller hydrophobic core makes LPS membranes very sensitive to the choice of equilibration protocol. In this work, we show that the protocol successfully used to equilibrate phospholipid bilayers when applied to complex lipopolysaccharide membranes occasionally leads to a small expansion of the simulation box very early in the equilibration phase. Although the use of a barostat algorithm controls the system dimension and particle distances according to the target pressure, fluctuation in the fleeting pressure occasionally enables a few water molecules to trickle into the hydrophobic region of the membrane, with spurious solvent buildup. We show that this effect stems from the initial steps of NPT equilibration, where initial pressure can be fairly high. This can be solved with the use of a stepwise-thermalization NVT/NPT protocol, as demonstrated for atomistic MD simulations of LPS/DPPE and lipid-A membranes in the presence of different salts using an extension of the GROMOS forcefield within the GROMACS software. This equilibration protocol should be standard procedure for the generation of consistent structural ensembles of charged glycolipids starting from atomic coordinates not previously pre-equilibrated. Although different ways to deal with this issue can be envisioned, we investigated one alternative that could be readily available in major MD engines with general users in mind.

## 1. Introduction

Molecular simulation methods provide the framework to bridge microscopic length and timescales to the macroscopic experimental world. These methods supply a precise route to compute thermodynamic and statistical properties, which can then be associated with molecular motions, structure, and function. Molecular dynamics (MD) simulations and variations are the most important computational techniques used to describe time-dependent properties of molecular systems [1], play an important role in the understanding of the physicochemical properties, structures, and functions of molecular systems, and are enablers of predictive molecular design. MD is also conceptually straightforward, simply requiring a particle-based description of the system of interest and the deterministic propagation of position, velocity, and force by employing a finite timestep to generate a collection of time-evolving configurations.

However, it is common sense that the devil is in the detail. Despite the consensus within the computational chemistry community regarding the most (and least) reliable algorithms, implementations, and simulation protocols underlying the MD method, system-specific issues are rather common in molecular simulations, as exemplified by a recent round-robin assessment of “simple” alkanes [2]. Regrettably, the increasingly larger scale computing resources are not sufficient to ensure appropriate sampling of the desired phase-space or accurate estimation of the uncertainties associated with a given molecular simulation protocol [3,4,5,6]. Another layer of intricacy lies in the adequacy of potential functions to accurately represent chemically complex systems. Lipopolysaccharides (LPS) are one class of complex materials for which not only simulations over longer timescales and larger spatial scales are required, but also the inaccurate treatment of both weak interactions and long-range interactions is detrimental [7,8]. Therefore, LPS membranes are expected to be particularly influenced by small variations in atomic parameters and schemes to mitigate cutoff errors in molecular simulations [9,10].

An illustrative example was recently provided for the effect of incorrect parametrization of the protonation state of LPS [11]. The representation of LPS phosphate groups as deprotonated (i.e., charge of −2*e* per phosphate) resulted in less compact LPS membranes [12]. This issue was compounded by the choice of parameter set for the ions, which led to LPS simulations with typically larger areas per lipid for divalent ions compared to monovalent ones, in contrast to experimental measurements [13] and previous atomistic simulations with different forcefields [14,15,16,17]. However, this attests to the fact that the adequate treatment of ions is the everlasting Achilles’ heel of classical forcefields [18]. Although more experienced simulators should be mindful of this, in practice, many users are unaware of it [19]. The impact of the issue reported by Rice and coworkers on the reliability of previous simulations of LPS membranes prepared via the webserver is unclear. However, this is a cautionary tale regarding the difficulties underlying molecular simulations of this chemically complex class of glycolipids.

We would like to contribute to the efforts attempting to improve the reliability of MD simulations of glycolipid aggregates through the assessment of equilibration protocols on the stability of LPS and lipid-A membranes. We chose to use the Berendsen thermostat and barostat due to their prevalence of use in molecular simulations [6], despite known artefacts associated with these algorithms [19]. However, we ascertained that the same equilibration issues occurred with the use of the Parrinello–Rahman barostat and the Nose–Hoover thermostat. We observed that the protocol successfully used to equilibrate phospholipid bilayers [20,21,22,23,24,25,26,27,28,29,30] when applied to glycolipid membranes using an extension of the GROMOS force-field [31] for LPS [16,32,33,34] and GROMACS v.2016.4 [35] occasionally allowed a few water molecules to trickle into the hydrophobic region of the membrane. Although neutron diffraction measurements [13] and atomistic MD simulations [14,15] showed that LPS membranes are greatly hydrated, when this behavior is associated with high pressure in the early phase of the equilibration, it leads to destabilization of the membrane. In this work, we identify the source of the leaky membrane effect in the equilibration protocol and recommend the inclusion of a short NVT phase before NPT equilibration when starting MD simulations of charged glycolipids from atomic coordinates not previously pre-equilibrated, ensuring consistent structural ensembles for the simulated glycolipid membranes, as discussed hereafter.

## 2. Results and Discussion

**Figure 1:**
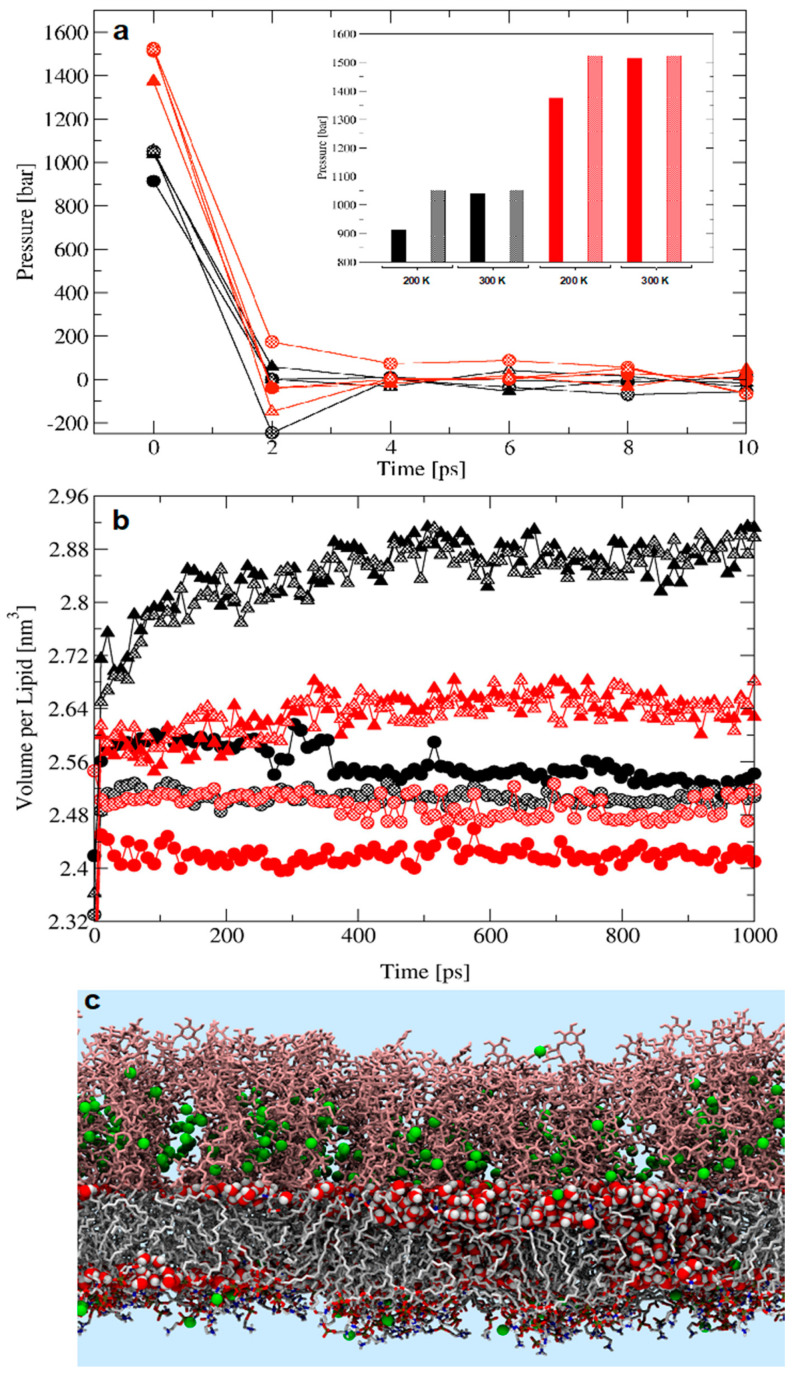
The effect of temperature, barostat coupling constant, and long-range electrostatic treatment on the (**a**) initial pressure and (**b**) V_L_ for lipopolysaccharide (LPS)/DPPE bilayers equilibrated under NVT/NPT conditions. The two long-range electrostatics approximations are shown in black (PME) and red (RF). The barostat coupling constants are represented by filled (0.1 ps) or dotted (1.0 ps) symbols and bars. Simulations performed at 200 K and 300 K are shown in circles and triangles, respectively. The inset shows pressure averages at the first step of the NPT phase for direct comparison. (**c**) Representative configuration of the LPS/DPPE bilayer with the largest value of volume per lipid (dotted black triangles). Only water molecules inside the hydrophobic region of the membrane are shown and near clipping of LPS molecules was applied to improve visualization. Ca^2+^ is shown in green and oxygen and hydrogen in water molecules are in red and white, respectively. Symbols correspond to lps1 (dotted black circles), lps2 (filled black circles), lps3 (dotted red circles), lps4 (filled red circles), lps5 (dotted black triangles), lps6 (filled black triangles), lps7 (dotted red triangles), and lps8 (filled red triangles).

It was observed that equilibration of glycolipid membranes from scratch using NPT-only conditions led to a small expansion of the simulation box very early in the equilibration. Although this behavior was often inconsequential, occasionally it allowed for the fast entry of a few water molecules into the hydrophobic region of the membrane with spurious solvent buildup in that region (Figure 1). This behavior is hereafter referred to as the leaky membrane effect. In the following discussion, we identify the main factor accountable for the effect through multiple short simulations of (very charged) LPS/DPPE membranes. The choice of benchmark system was justified by the highly charged nature and complex hydration of LPS membranes so that it was very responsive to subtleties in the equilibration protocol. Subsequently, we demonstrate the appropriateness of a modified protocol to generate compatible structural ensembles from a series of longer MD simulations of lipid-A membranes in the presence of different salts.

### 2.1. The Matter with Standard Equilibration Protocols for Glycolipid Membranes

The leaky membrane effect was observed for all LPS/DPPE and lipid-A membranes equilibrated under NPT-only conditions, regardless of the choice of barostat and thermostat. Therefore, this protocol is not recommended for simulations of highly charged glycolipid membranes using the atomic parameters, simulation conditions, and MD engine described in the methods section (Figure S3). On the other hand, preceding the NPT equilibration by a short equilibration under NVT conditions provided a general, yet efficient solution. However, this may also lead to the leaky membrane effect in glycolipid membranes if certain setup choices are made. Hence, variations in the NVT/NPT protocol were used to equilibrate the LPS/DPPE membrane (Table 1), starting from the same configuration in all simulations. The leaky membrane effect could be traced back to the first step of the equilibration where the initial pressure was very high, more so at higher temperatures (Figure 1). Context around this statement is given below.

The pressure of an isolated system is composed of the kinetic energy and the intermolecular forces between particles, i.e., nonbonded interactions. Intramolecular interactions contribute negligibly to the system total pressure [36,37]. In MD simulations, the intermolecular forces contribute to the total pressure according to the virial Equation (1) [38].

(1) $$P=\frac{1}{V}\;\left(\underset{i}{\sum }m_{i}v_{i}v_{i}^{T}+\underset{i<j}{\sum }r_{ij}F_{ij}^{T}\right)$$

where **P** is the pressure tensor, V is the volume, m_i_ is the mass of the ***i*^th^** particle, ***v_i_*** is the velocity vector of the ***i*^th^** particle, ***r_ij_*** is the distance between the ***i*^th^** and ***j*^th^** particles (or the nearest image of particle ***j***), and ***F_ij_*** is the force exerted on the particle ***i*** by the particle ***j*** due to pair-additive potentials. The pressure can be controlled by the means of a barostat algorithm, which scales the system dimension and particle distances so that the former increases or decreases as the pressure fluctuates above or below a target pressure, respectively. An example of such algorithm is the Berendsen barostat, which uses an external constant pressure bath (Equations (2)–(4)) [38].

(2) $$\mu =1-\frac{\beta \Delta t}{3\tau_{p}}\;\left(P_{o}-P\right)$$

(3) $$r_{i}^{\prime }=\mu r_{i}$$

(4) $$V^{\prime }=\left(\det\mu \right)V$$

where ***μ*** is the scaling matrix, ***β*** is the isothermal compressibility (which can be treated as a tensor), *Δt* is the timestep, *τ_p_* is the time constant of the coupling, **P_o_** is the target pressure, ***r′_i_*** is the rescaled position of ***i^th^*** particle, and V′ is the rescaled volume of the system. For systems with semi-isotropic pressure coupling, x and y axes are scaled isotropically, while the z-axis is scaled independently. The larger the pressure deviates from the target value, the greater the rescaling of the system size. Hence, if the initial configuration of a MD simulation in the NPT ensemble has regions of abnormally high forces between particles, significant pressure deviation from the target value is expected. As the scaling factor is applied to all particles in the system, drastic system size rescaling occurs, which generates nonrealistic configurations. In the case of highly charged systems (e.g., LPS membranes), where interparticle forces are of great magnitude, these nonphysical configurations may occur more often.

After the NVT phase at 100 K, the pressure in LPS/DPPE simulations remained high during the first step of the NPT phase (Figure 1). Because the pressure is calculated as the contributions of the kinetic energy and the virial (Equation (1)) defined by the intermolecular forces, the pressure is directly associated with a given configuration. Hence, the initial high pressure was linked to the final configuration sampled in the previous NVT phase and used in the subsequent NPT phase of the equilibration. The system pressure is also influenced by the temperature and the treatment of long-range electrostatics (Figure 1). Comparatively, the effect of the temperature on the pressure is small because it is controlled by the use of a thermostat. The influence of the choice of long-range electrostatics approximation is also noticeable as it derives from method-inherent differences in the calculation of electrostatics forces between particles beyond a given cutoff (Figure 1), exerting an addictive effect on the intermolecular forces and thus on the virial. The use of a barostat ensures that the anomalous pressure rapidly converges to the reference value (within less than 10 ps) in all simulated systems (Figure 1). In our simulations, the treatment of long-range electrostatics did not lead to significant differences in pressure values after 10 ps of equilibration (Figure 1a), consistent with previously published simulations of LPS/DPPE membranes using Reaction Field (RF) and Particle-Mesh Ewald) PME approximations to treat long-range electrostatics [39].

**Figure 2:**
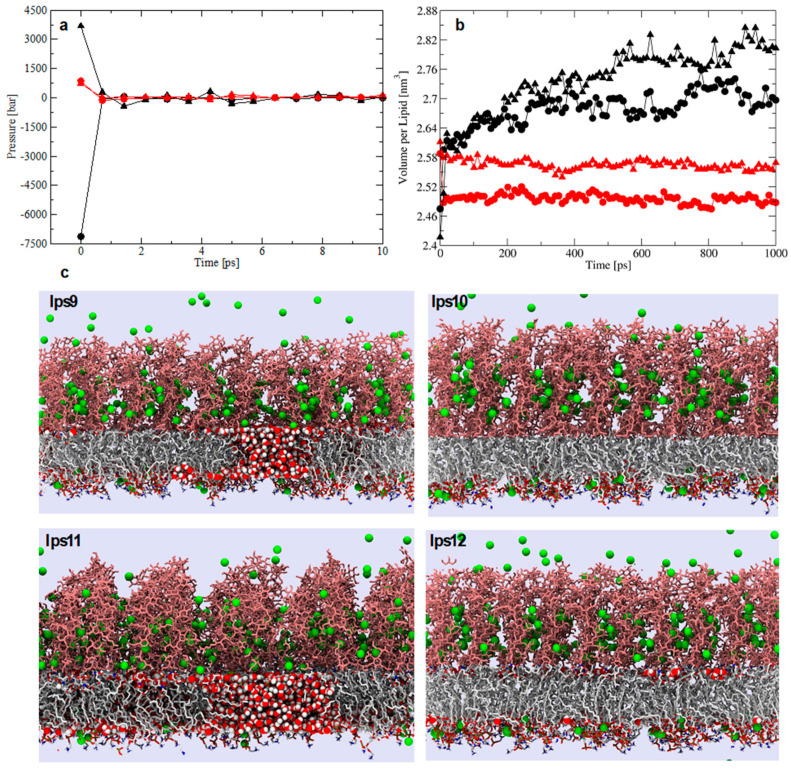
The effect of the equilibration protocols (NPT-only and stepwise thermalization NVT/NPT protocols), barostat (Berendsen and Parrinello–Rahman), and thermostat (Berendsen and Nose–Hoover) on the (**a**) initial pressure and (**b**) molecular volume of LPS/DPPE bilayers. Symbols are NPT-only (black) and stepwise-thermalization NVT/NPT (red) protocols, Berendsen/Berendsen (circles), and Parrinello–Rahman/Nose–Hoover (triangles) barostats/thermostats, respectively. (**c**) Representative conformations from lipid-A simulations using the single-temperature NVT/NPT protocol. Systems are lps9 (black circle), lps10 (red circle), lps11 (black triangle), and lps12 (red triangle). Only water molecules inside the hydrophobic region of the membrane are shown and near clipping of LPS molecules was applied to improve visualization.

Although the rapid pressure convergence suggested that the simulations underwent normal equilibration, some of the membrane configurations associated with the initial high-pressure values in the NPT phase of NVT/NPT equilibration underwent volume expansion (Figure 1b,c). These alterations in membrane packing could be probed through time-dependent variation of the volume per lipid (Figure 1b,c). While the pressure converged to the reference value, the corresponding volume per lipid for the LPS/DPPE membranes differed significantly from each other, even with a common initial configuration for all the simulations. The increase in the volume per lipid could be influenced, to a greater or lesser extent, by increase in temperature (200 K versus 300 K), choice of long-range electrostatics treatment (RF versus PME), and larger barostat coupling constants (0.1 ps versus 1 ps) (Figure 1a,b). Moreover, increase in volume per lipid also occurred consistently for the NPT-only equilibration, regardless of the thermostat/barostat tested (Table 1), ultimately leading to spurious water penetration in the hydrophobic region of the membrane (Figure 2). Therefore, the equilibration issue appears to be inherent to the chemical nature of charged glycolipid membranes rather than to previously reported artefacts associated with thermostat/barostat algorithms [40].

### 2.2. Validation of the Modified Equilibration Protocol for Glycolipid Membranes

Comparison of the three equilibration protocols for simulation of the LPS/DPPE reference systems demonstrated that NPT-only invariably yielded leaky membranes, while the single-temperature NVT/NPT often (but not always) yielded leaky membranes and the stepwise-thermalization NVT/NPT consistently yielded stable membranes (Figure 2). Therefore, we recommend the use of the stepwise NVT/NPT equilibration protocol with low values for the pressure coupling constant when using the Berendsen barostat (Figure 2) to damp lower initial pressures and avoid spurious conformations leading to inconsistent, and incorrect, structural ensembles. We also performed simulations of the membranes using the NPT-only protocol and different pressure coupling times for the z-axis and the xy-plane. This alternative was expected to allow for the equilibration of the polysaccharide chains in the water phase while retaining the intended area per lipid until the system was ready to go full-NPT. However, it also led to water trickling and buildup in the membrane (Figure S4), possibly because of the assumption that the initial configuration of the membrane had the correct area per lipid, which was intentionally not ensured in our initial configurations. Indeed, equilibration protocols should be robust enough to bring the area per lipid of a bilayer to equilibrium. Hereafter, we assess the responsiveness of structural properties commonly used in the analysis of MD simulations of membranes to distinguish between leaky and stable lipid-A membranes over timescales of 400 ns after equilibration.

**Figure 3:**
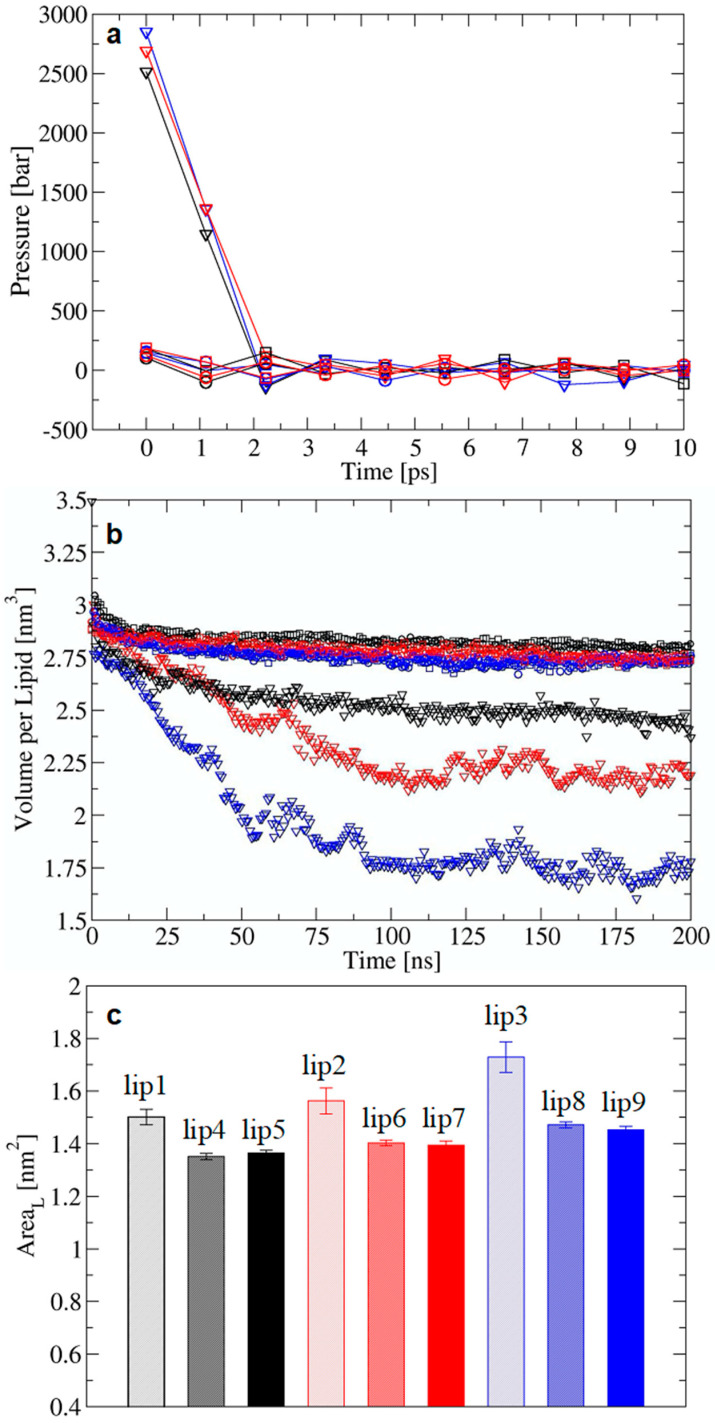
The effect of the equilibration protocols on the initial pressure and structural properties of lipid-A membranes in the presence of Al^3+^ counterions without additional salts (black), 150 mM of AlCl_3_ (red), and 150 mM of NaCl (blue). (**a**) Initial pressure for equilibration with NPT-only (triangles) and stepwise-thermalization NVT/NPT using reaction field (squares) or PME (circles). (**b**) Volume per lipid, V_L_. (**c**) Area per lipid, A_L_. Averages were calculated over the last 100 ns of simulation.

**Figure 4:**
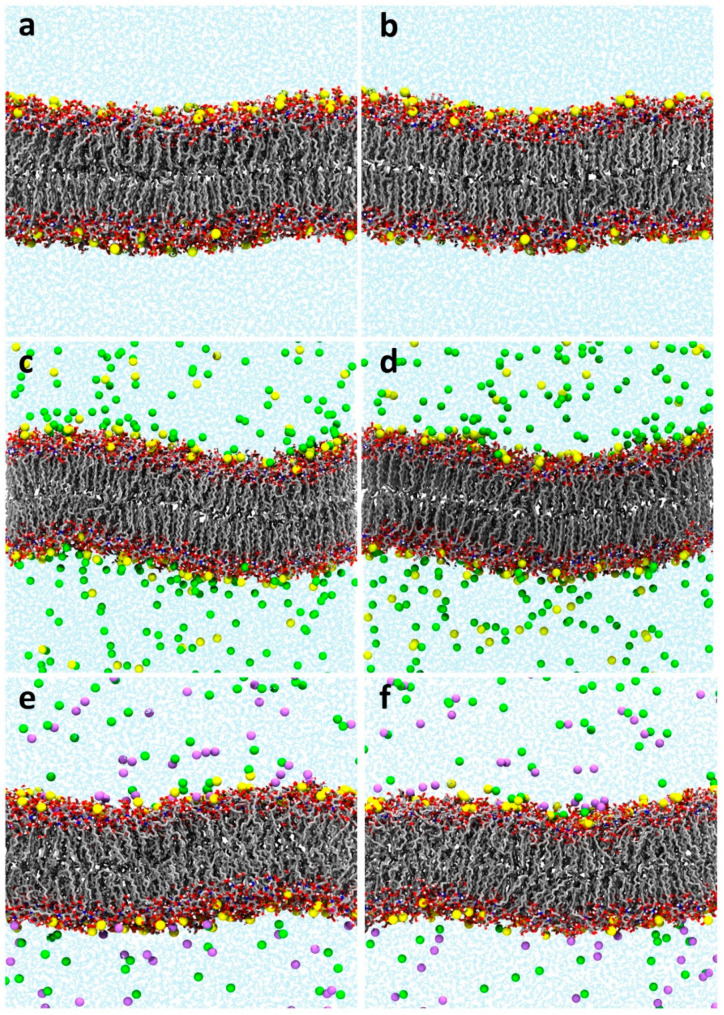
Representative conformations from MD simulations of lipid-A membranes in the presence of Al^3+^ counterions using the stepwise-thermalization NVT/NPT protocol and two long-range electrostatic approximations. Lipid-A bilayers without (**a**,**b**) addition of salts, (**c**,**d**) with addition of 150 mM of AlCl_3_, and (**e**,**f**) with addition of 150 mM NaCl. Long-range electrostatic interactions were approximated using reaction-field (first column) or particle-mesh Ewald (second column). Simulations were run in duplicate for 400 ns after the equilibration phase with the stepwise thermalization protocol. Al^3+^, Na^+^, and Cl^−^ are shown in yellow, magenta, and green van der Waals spheres, respectively.

**Figure 5:**
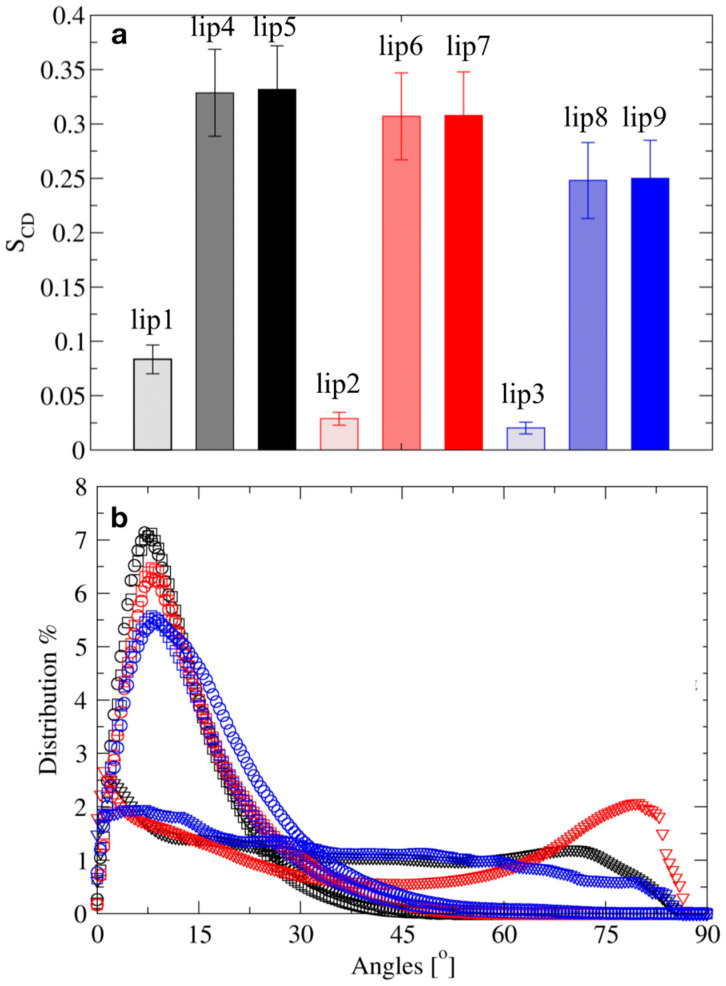
The effect of the equilibration protocols on the dynamical properties of the lipid-A membranes in the presence of Al^3+^ counterions without additional salts (black), 150 mM of AlCl_3_ (red), and 150 mM of NaCl (blue). Equilibration with NPT-only (triangles) and stepwise-thermalization NVT/NPT using the reaction field (squares) or PME (circles) approximations. (**a**) Average deuterium order parameters S_CD_ for acyl chains. (**b**) Distribution of the time-averaged surface curvature angle, S_C_. Averages were calculated over the last 100 ns of simulation.

We applied the stepwise-thermalization NVT/NPT protocol to a series of Al^3+^-containing lipid-A membranes in different concentration regimes to contrast with the NPT-only protocol (Table 2). As seen for the LPS/DPPE systems, the high initial pressure at the beginning of the NPT phase (and after the NVT phase) was a signature for potential membrane instability that may have led to the water leakage and buildup in the hydrophobic region observed in the lipid-A membrane simulations. The outcome was the same as for LPS/DPPE membranes, i.e., the NPT-only often yielded leaky membranes and the stepwise-thermalization NVT/NPT consistently yielded stable membranes (Figure 2). Hence, we also assessed the structural properties of the lipid-A membranes after the equilibration using the two protocols to ensure the generated structural ensembles were stable at longer timescales. MD simulations of Al^3+^-containing lipid-A membranes in 0 mM, 150 mM of AlCl_3_, and 150 mM of NaCl were performed in duplicate for 400 ns (Table 2). Although the initial high pressure in the NPT-only protocol rapidly converged to the target pressure, it did lead to a decrease in the membrane molecular volume (Figure 3). Conversely, the stepwise-thermalization NVT/NPT protocol generated structural ensembles which were stable at the nanosecond timescale (Figure 4). Furthermore, there were no significant differences between the structural properties calculated from simulations using the two long-range electrostatic approximations (Figure 3 and Figure 5) as long as the optimal conditions associated with the respective approaches were enforced.

The area per lipid (A_L_) for the simulated lipid-A membranes did not reflect the major structural differences associated with the use of two equilibration protocols (Figure 3). On average, the A_L_ differed by ca. 0.2 nm^2^ between stable and leaky membranes (Figure 3), which was within the same value for the difference between A_L_ for lipid-A membranes with different representations of protonation states [11]. Experimentally, A_L_ can differ between the gel and liquid crystalline states of a given LPS chemotype in 0.03 to 0.3 nm^2^ depending on the experimental setup [41,42]. Therefore, the increase in molecular volume due to the leaky membrane effect may not be easily detected through A_L_ values, even after full equilibration. Although A_L_ is widely used to compare membrane simulations to experimental data, caution should be exerted in cases where local changes may not reflect on A_L_, which is averaged over the total number of lipids in the membrane. This issue may be more troublesome for LPS and lipid-A due to the high number of acyl chains per molecule, and potentially larger dispersion of average A_L_ values.

On the other hand, the carbon–deuterium order parameter (S_CD_) revealed a clear distinctive pattern between simulations generated with the two equilibration protocols (Figure 5a). The S_CD_ values decreased severely for the systems equilibrated with the NPT-only protocol. The salt-free simulations displayed a reduction in S_CD_ from ca. 0.33 to c 0.08, while the decreases for simulations in saline solutions of AlCl_3_ and NaCl were in the range of ca. 0.25 to 0.02. and ca. 0.30 to 0.02, respectively. The abnormally small values of S_CD_ values indicated that the acyl chains rotated almost freely in the lip1, lip2, and lip3 simulations, implying a severe loss of structure. This was confirmed through the analysis of the distribution frequencies for the surface curvature angle (Figure 5b). The stepwise-thermalization NVT/NPT protocol generated structural ensembles for which the surface angle distributions increased steeply for low angle values, reaching the maximum at ca. 7.5°. The probability of curvature angles with values equal to or larger than 30° was considerably low, indicating a “well-behaved” bilayer and occurrence of only low-amplitude bilayer fluctuations (Figure 5b). In contrast, the NPT-only protocol yielded structural ensembles with angle distribution maximum peaks shifting from low to high curvature values (>30°), indicating loss of lamellarity of the bilayer.

## 3. Materials and Methods

MD simulations were performed for two different glycolipids. One system was composed of LPS and 1,2-dipalmitoyl-*sn*-glycerol-3-diphosphatidylethanolamine (DPPE) arranged in separate leaflets, and the other type corresponded to diphosphorylated lipid-A bilayers. The LPS/DPPE bilayers were composed of 100 LPS and 250 DPPE molecules, so that the total numbers of acyl chains were equal between the two leaflets (Table 1), whereas the lipid-A bilayers were made of 162 lipids equally distributed in a 9 × 9 arrangement per layer (Table 2). An extension of the GROMOS force field was used [32,34], which builds up from previously validated glucosamine residues and encompasses standard bonded parameters between the sugar moiety and the acyl chains compatible with the GROMOS 53A6 lipid parameter set [43,44].

This parameter set was previously tested for LPS and the lipid-A bilayers [16,34,39]. Ca^2+^, Na^+^, and Cl^−^ ions parameters were also taken from the GROMOS 53A6 forcefield, whereas interaction potentials for the Al^3+^ ion, an important vaccine adjuvant, were taken from [45]. By default, the classical treatment of these ions neglected electronic effects. The SPC water model and periodic boundary conditions were used throughout the simulations [46]. A total of 51,365 water molecules were added to the LPS–DPPE bilayers and 40,172 molecules to the lipid-A ones. LPS and lipid-A at neutral pH have total charges of −8*e* and −2*e*, respectively. The charges were neutralized through the addition of 400 Ca^2+^ and 108 Al^3+^ counterions to the former and latter systems, respectively. The simulations of Al^3+^-containing lipid-A membranes were performed at three different salt concentration regimes, namely, no added salt (except for the Al^3+^ counterions required to ensure the electroneutrality of the system), 150 mM AlCl_3_, and 150 mM NaCl (Table 2). The salt concentration was calculated based on the solvent box volume, excluding the bilayer volume.

Initial configurations of the membrane were built by replicating previously equilibrated membranes of 4 × 4 LPS/10 DPPE and 4 × 4 lipid-A molecules. These pre-equilibrated membranes were generated by placing geometry-optimized, randomly rotated, lipid units on a regularly spaced grid, an approach similar to the one originally proposed in [47]. During the solvation phase of this setup, the van der Waals radii of atoms in the acyl chains were temporarily increased to ensure that water molecules were added exclusively in the carbohydrate region. Neutralizing counterions were placed in the vicinity of the negatively charged groups of the glycolipid unit previous to the addition of concentration salts. After replication of the pre-equilibrated membranes to create the simulated systems listed in Table 1 and Table 2, all systems were geometry-optimized using the steepest descent algorithm without constraints for 5000 steps, subsequently followed by the equilibration phase. Snapshots of the initial configuration of LPS/DPPE and lipid-A membranes used in the simulations are presented in the supplementary information (Figures S1 and S2). Three equilibration protocols were considered based on the simplicity of use with the GROMACS software, to which our atomic parameters were originally ported. In the NPT-only protocol, the temperature was kept at 300 K, starting from an initial distribution at 10 K, via the Berendsen thermostat [38], with a coupling constant of 0.4 ps. The pressure was also kept constant via the Berendsen barostat [38] in a semi-isotropic scheme at 1 bar with a coupling constant of either 0.1 ps or 1.0 ps and compressibility of 4.5 × 10^−5^ bar^−1^. In the single-temperature NVT/NPT protocol, a 500 ps relaxation was performed in the NVT ensemble at 300 K using the Berendsen thermostat [38], with a coupling constant of 0.4 ps. Upon temperature convergence, the simulation conditions were shifted from NVT to NPT using the same conditions as in the NPT-only protocol. The stepwise-thermalization NVT/NPT protocol was the same as the single-temperature one, except that the temperature in the NPT phase increased in a stepwise manner from 100 K to 300 K. Furthermore, we replicated the LPS/DPPE benchmark simulations but used the Nose–Hoover thermostat [48] (coupling constant of 0.5 ps) and the Parrinello–Rahman barostat [49] (coupling constant of 5.0 ps). These latter simulations intended to ascertain that the observed equilibration issue was not related to known deficiencies of weak-coupling thermostats, but rather inherent to the chemical nature of the lipopolysaccharide membranes [6]. We did not address thermostat-related artefacts extensively reported in the last 20 years [6,40]. However, because weak-coupling thermostats remain the most popular choice by general users of biomolecular simulation engines [6], we sought to conceive a protocol that would also be effective when using the Berendsen thermostat.

After the equilibration phase, duplicates were run in the NPT ensemble for 400 ns using the leapfrog algorithm, with a timestep of 2 fs. Hydrogen bond lengths within the solute were constrained using the LINCS algorithm [50]. The geometry of the water molecules was constrained using the SETTLE algorithm [51]. Pressure was kept at 1 bar using a semi-isotropic scheme and the Berendsen barostat [38], with the pressure coupling frequency adjusted to 0.4 ps. A cutoff of 1.4 nm for both electrostatics and van der Waals interactions was used throughout the equilibration and production phases. Two long-range electrostatic schemes were used during the production phase to ensure that the observed behavior was independent of the approximation used to treat the interactions. The generalized reaction-field (RF) [52], with a relative dielectric permittivity constant of 66 [53], was conventionally used with the GROMOS forcefield and the particle mesh Ewald summation (PME) [54]. In the PME simulations, the charges were projected onto a 0.16 nm grid using a cubic interpolation for the calculation of long-range electrostatic interactions in reciprocal space. Pair lists were updated every 10 fs with the use of RF. The Verlet cutoff scheme [55] was applied to the PME simulations, and as result of its implementation in GROMACS v.2016.4 [35], the nstlist and rlist values were code-adjusted from 5 to 40 and from 1.4 to 1.422 nm, respectively. The full simulations of the LPS-DPPE membranes using the NVT/NPT protocol were published ref [39]. MD simulations were performed using GROMACS v.2016.4 [35] and analyzed using GROMACS v.2016.4 [35] and SuAVE software [56].

## 4. Conclusions

In this work, we show that the equilibration protocol successfully applied to phospholipid membranes may lead to a small expansion of the simulation box very early in the equilibration phase when applied to complex lipopolysaccharide membranes. Since the use of a barostat algorithm scales the system dimension and particle distances so that the former increases or decreases as the pressure fluctuates above or below a target pressure, the anomalous pressure is not easily noticed. However, the box expansion associated with this fleeting pressure fluctuation occasionally enabled a few water molecules to trickle into the hydrophobic region of the membrane with spurious solvent buildup. This leaky membrane effect could be ascertained to the initial steps of the NPT equilibration, where initial pressure can be fairly high, more so if not combined with a stepwise increase in temperature. We recommend users to adhere to a double-step equilibration protocol shown to generate consistent structural ensembles for complex glycolipid membranes, even when starting from less than optimal initial configurations of the systems. The consistency of the two-step versus single-step protocols was demonstrated for MD simulations of LPS/DPPE and lipid-A membranes in the presence of different salts. The use of the stepwise-thermalization NVT/NPT protocol is recommended when starting atomistic MD simulations of charged glycolipids using the extension of the GROMOS force-field [31] for LPS [16,32,33,34] and GROMACS v.2016.4 [35] from atomic coordinates not previously pre-equilibrated.

## Figures and Tables

**Table 1:** Molecular dynamics (MD) simulations of LPS/DPPE membranes using the NVT/NPT equilibration protocol and different barostats, thermostats, and long-range electrostatic (LRE) approximations. Pressure (τ_p_) and temperature (τ_T_) coupling constants in ps. Simulations were performed in duplicate.

Systems	NVT Step	NPT Step	τ_p_	τ_T_	LRE
lps1	300 K	200 K	1.0	0.4	PME
lps2	300 K	200 K	0.1	0.4	PME
lps3	300 K	200 K	1.0	0.4	RF
lps4	300 K	200 K	0.1	0.4	RF
lps5	300 K	300 K	1.0	0.4	PME
lps6	300 K	300 K	0.1	0.4	PME
lps7	300 K	300 K	1.0	0.4	RF
lps8	300 K	300 K	0.1	0.4	RF
lps9	-	100 K→200 K→300 K	0.1	0.4	PME
lps10	100 K	100 K→200 K→300 K	0.1	0.4	PME
lps11 *	-	100 K→200 K→300 K	5.0	0.5	PME
lps12 *	100 K	100 K→200 K→300 K	5.0	0.5	PME

* All simulations were performed using the Berendsen thermostat and barostat, except lps11 and lps12 simulations, which were performed using the Parrinello–Rahman barostat and the Nose–Hoover thermostat. PME (Particle-Mesh Ewald). RF (Reaction Field).

**Table 2:** MD simulations of lipid-A membranes in the presence of Al^3+^ counterions treated via different equilibration protocols, long-range electrostatic (LRE) approximations, and concentration regimes (no additional salt, 150 mM of NaCl, 150 mM of AlCl_3_). A pressure coupling constant τ_p_ of 0.4 ps was applied to during the production phase for the simulations. The simulations were performed in duplicate.

Systems	Protocol	LRE	Ions		
			Al^3+^	Na^+^	Cl^−^
lip1	NpT	RF	108	0	0
lip2	NpT	RF	219	0	333
lip3	NpT	RF	108	111	111
lip4	NVT/NpT	RF	108	0	0
lip5	NVT/NpT	PME	108	0	0
lip6	NVT/NpT	RF	219	0	333
lip7	NVT/NpT	PME	219	0	333
lip8	NVT/NpT	RF	108	111	111
lip9	NVT/NpT	PME	108	111	111

## Supplementary Materials

The following are available online, Figure S1: Initial configuration of the LPS + DPPE membrane used in the simulations indicated in Table 1; Figure S2: Initial configuration of the lipid-A membrane used in the simulations indicated in Table 2; Figure S3: Representative conformations from MD simulations of the lipid-A membranes in the presence of Al^3+^ counterions using the NPT-only protocol (first column) or stepwise-thermalization NVT/NPT protocol (second column); Figure S4: Representation of 50-ns conformations from MD simulations of the LPS/DPPE membrane equilibrated under NPT-only conditions with different pressure coupling along the z-axis and the xy-plane of the membrane. Compressibility of zero along the a) z-axis and b) xy-plane.
