# Supplementary material for: Out of Sight, Out of Mind: The Effect of the Equilibration Protocol on the Structural Ensembles of Charged Glycolipid Bilayers
Source: Molecules. 2020 Nov 4;25(21):5120. doi: 10.3390/molecules25215120 (PMC7663769; doi:10.3390/molecules25215120)
Supplement: Supplementary file 1 [file molecules-25-05120-s001.pdf]

molecules

Out of Sight, Out of Mind: The Effect of The Equilibration Protocol on the Structural Ensembles of Charged Glycolipid Bilayers

Andresa Messias, Denys E. S. Santos, Frederico J. S. Pontes, Filipe S. Lima, Thereza A. Soares

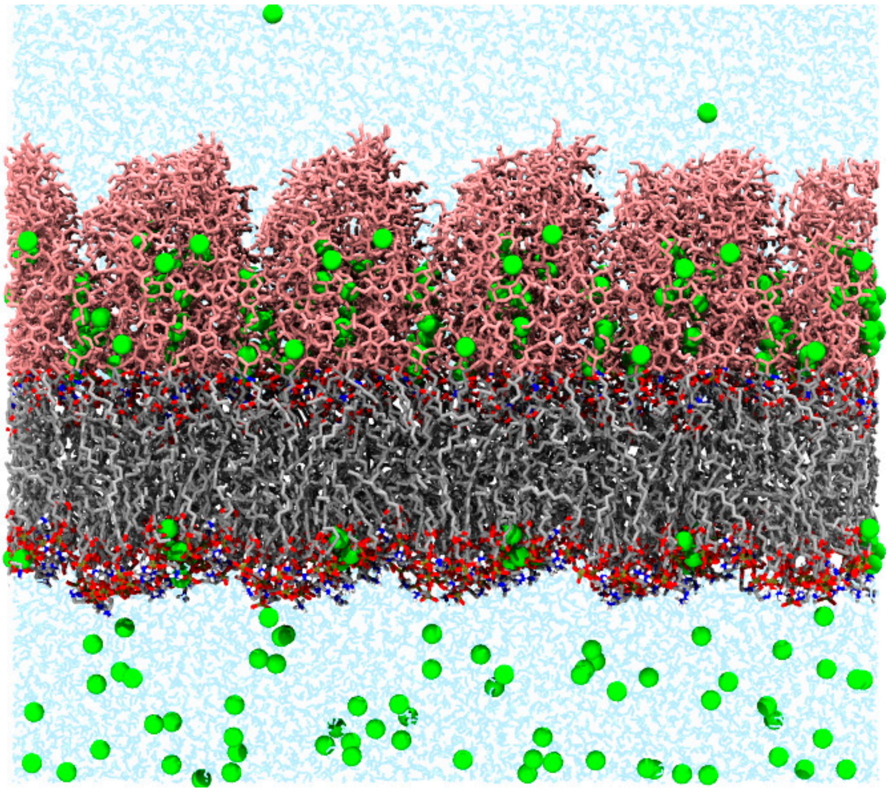

S1. Initial configuration of the LPS+DPPE membrane used in the simulations indicated in Table 1. Acyl chains, carbohydrate moieties, nitrogen and oxygen are represented in gray, pink, blue and red (respectively) sticks. Ca^{2+} are shown in green van der Waals spheres and water molecules are in cyan

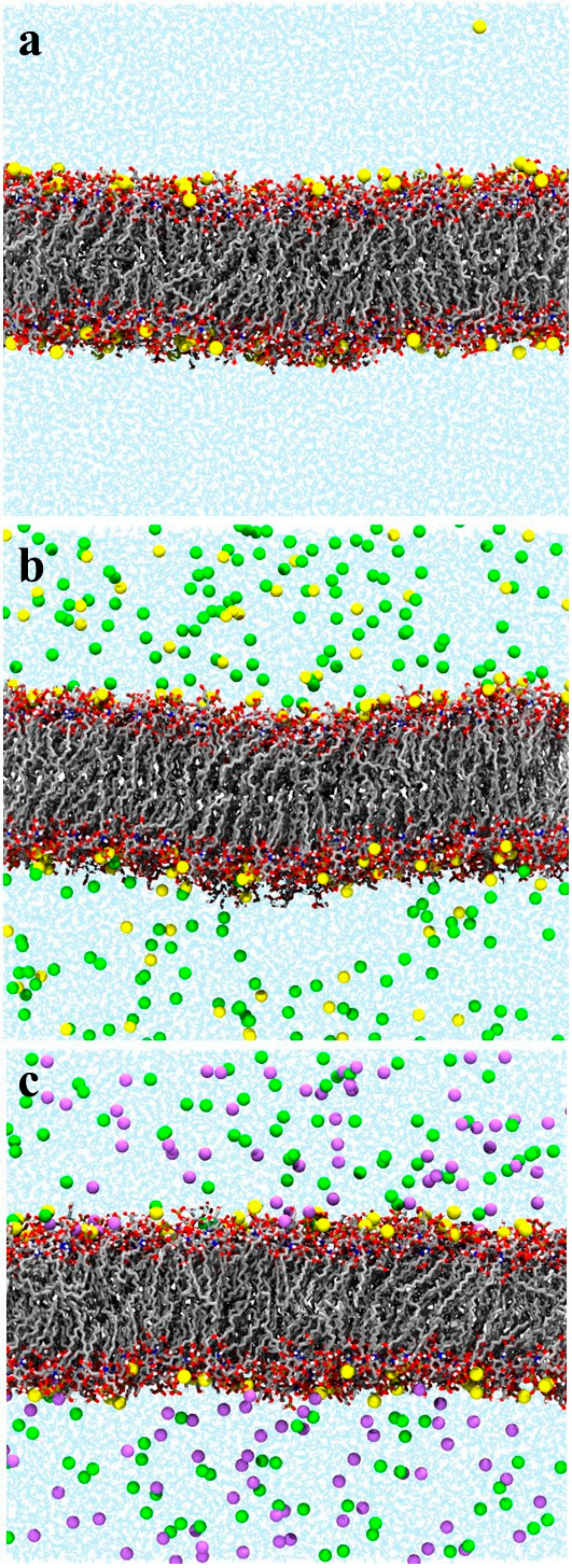

S2. Initial configuration of the Lipid-A membrane used in the simulations indicated in Table 2. Lipid-A bilayer (**a**) without addition of salts, (**b**) with addition of 150mM of $AlCl_3$ and (**c**) with addition of 150mM of NaCl. Acyl chains are represented in gray sticks. Al^{3+}, Na^+ and Cl^- are shown in yellow, magenta and green van der Waals spheres, respectively. Water molecules are in shown in cyan.

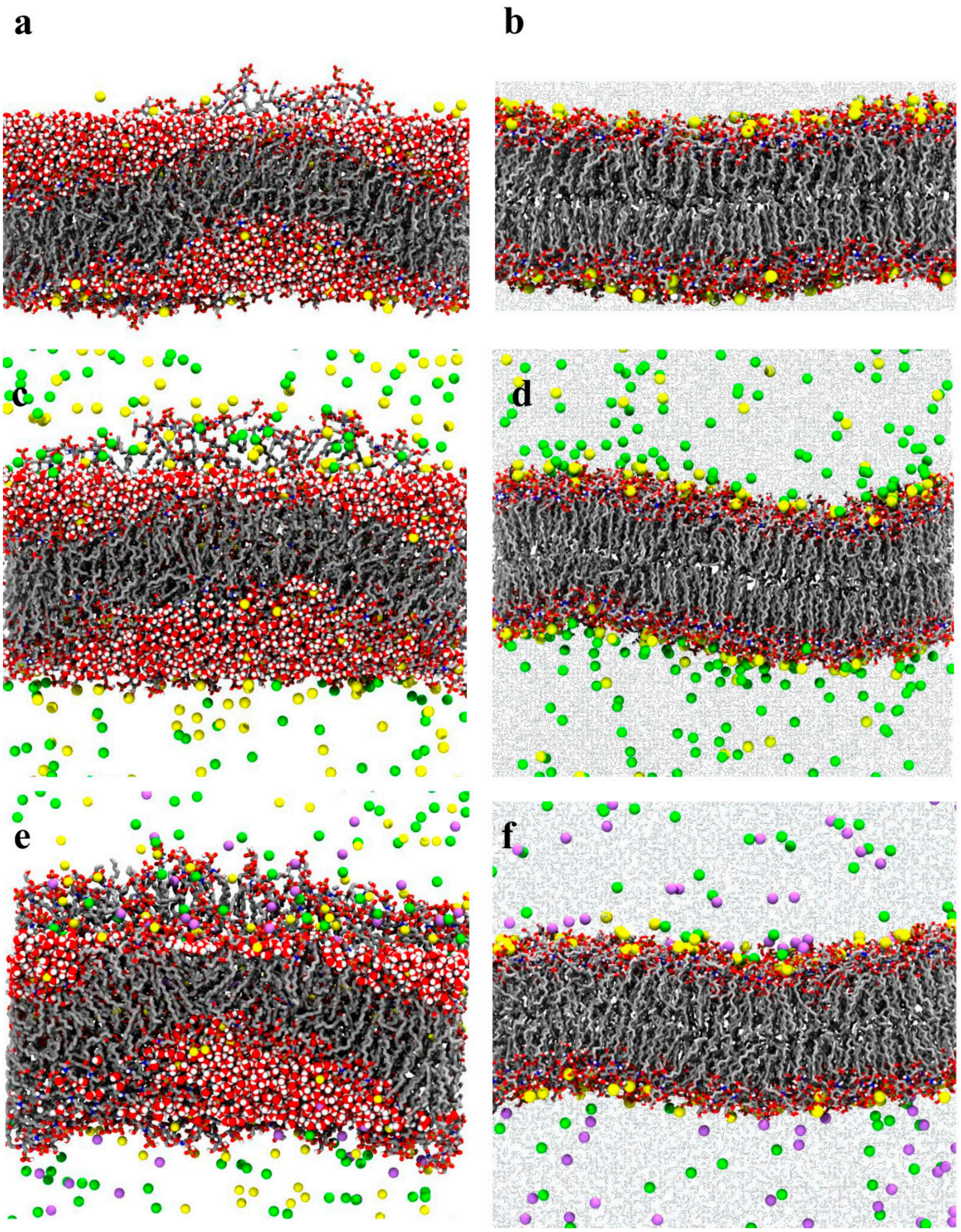

S3. Representative conformations from MD simulations of Lipid-A membranes in presence of Al^{3+} counterions using the NPT-only protocol (first column) or stepwise-thermalization NVT/NPT protocol (second column). **a-b.** Lipid-A bilayers without addition of salts, **c-d.** with addition of 150mM of AlCl3 and **e-f.** with addition of 150 mM of NaCl. Long-range electrostatic interactions were treated using reaction-field. Al^{3+}, Na^{+} and Cl^{-} are shown in yellow, magenta and green van der Waals spheres, respectively. Only water molecules near the membrane surface are shown for clarity in the first column.

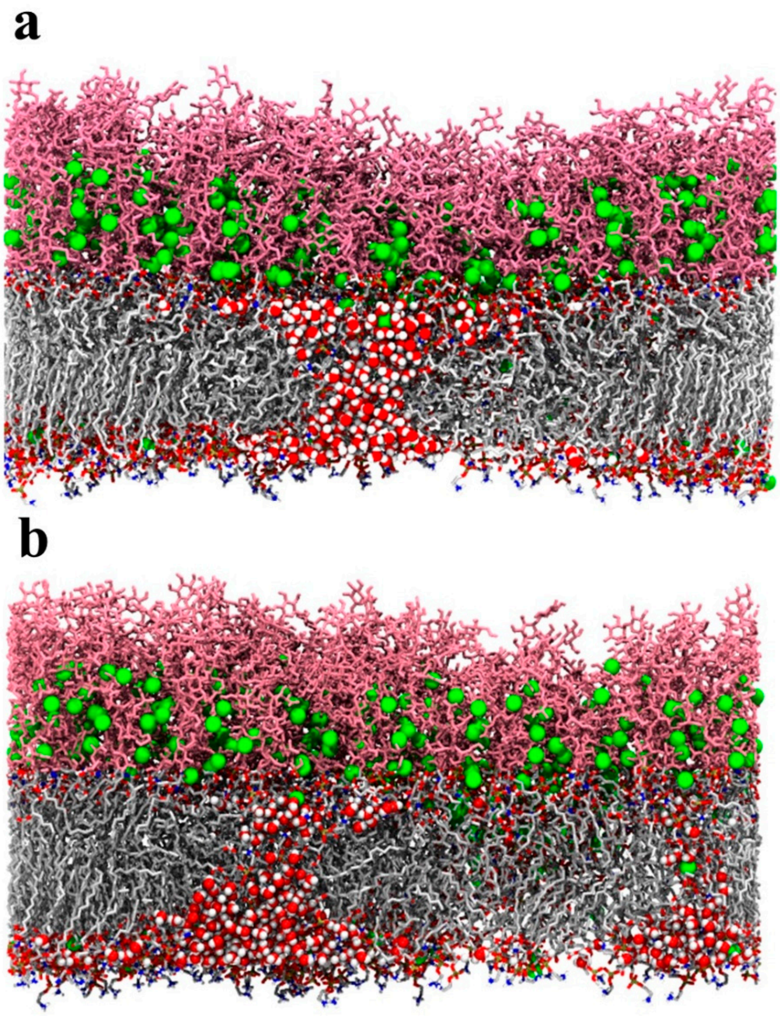

S4. Representation of 50-ns conformations from MD simulations of LPS/DPPE membrane equilibrated under NPT-only conditions with different pressure coupling along the z-axis and the xy-plane of the membrane. Compressibility of zero along the a) z-axis and b) xy-plane.
